# Decreased Clinical Severity of Pediatric Acute COVID-19 and MIS-C and Increase of Incidental Cases during the Omicron Wave in Comparison to the Delta Wave

**DOI:** 10.3390/v15010180

**Published:** 2023-01-07

**Authors:** Patrick O. Kenney, Arthur J. Chang, Lorna Krabill, Mark D. Hicar

**Affiliations:** 1 Jacobs School of Medicine and Biomedical Sciences, University at Buffalo, Buffalo, NY 14203, USA; 2 Oishei Children’s Hospital, Buffalo, NY 14203, USA; 3 Children’s Hospital & Medical Center, Omaha, NE 68114, USA

**Keywords:** COVID-19, MIS-C, pediatric hospitalizations

## Abstract

This study describes differences in clinical presentation in hospitalized children with acute COVID-19 and MIS-C between the Delta and Omicron (BA.1.1) waves in a tertiary children’s hospital. This retrospective cohort study with case adjudication of hospitalized children with SARS-CoV-2-positive testing or MIS-C diagnosis occurred during the Delta and Omicron waves, from August 2021 until February 2022. There were no differences noted by race, but both waves disproportionally affected black children (24% and 25%). Assigned by a three-person expert panel, incidental diagnoses were higher in the Omicron wave (34% versus 19%). Hospitalization rates of non-incidental cases were higher during Omicron (3.8 versus 5.9 per 1000 PCR-positive community cases). Respiratory-related admissions were prominent during Delta, while Omicron clinical presentations varied, including a high number of cases of croup and seizures. Length of stay and ICU use during Omicron was significantly less than Delta for MIS-C and acute cases. Estimation of vaccination efficacy for preventing hospital admissions was 85.1–91.7% in the early Omicron period. Our estimates suggest that a protective role for vaccination continues into the Omicron wave. The high rate of incidental cases during the Omicron wave should be considered when reviewing more cursory summative data sets. This study emphasizes the need for continued clinical suspicion of COVID-19 even when lower respiratory symptoms are not dominant.

## 1. Introduction

Data are emerging on the newest SARS-CoV-2 circulating variants. In population-based studies incorporating adults and children, the Omicron (BA.1) wave caused overall more hospitalizations and more ICU admissions than Delta [1]. This may be due to increased transmissibility. A Scottish study supports Omicron is less severe than Delta, and vaccination is still protective against Omicron [2]. A large study incorporating both inpatient and outpatient data supports that Omicron is less severe than Delta, with the severity difference most pronounced in those without prior immunity [3].

In studies including pediatric data, more children were hospitalized during Omicron [1,4,5]. When case severity was compared to admission rates rather than based on population, Omicron cases showed decreased markers of severity (ICU utilization, length of stay, and death) [6]. In particular, the diagnosis of croup being associated with Omicron variants has been noted, which could influence overall severity indicators [7,8].

During the Omicron waves, reports have also indicated decreased severity and decreased incidence of Multisystem Inflammatory Syndrome in Children (MIS-C) [9]. Data from Israel showed a 13-fold reduction in cases from Delta to Omicron [10]. Some small cohort studies suggest that the phenotypes are similar [11], however one study showed a trend to shorter length of stays [9], and another study supported less vasopressor use and ICU utilization [10]. Vaccination is protective against developing MIS-C in pre-Omicron studies [12] and seems to continue to protect children during Omicron from severe disease and hospitalization [13]. As vaccine usage in children 5–11 years of age was not available until midway into the Delta wave in November of 2021 [14], further analysis of vaccine protection is warranted.

In our clinical practice, we noted less severe MIS-C cases, a paucity of vaccinated admissions and a number of incidental cases of SARS-CoV-2 positivity during the Omicron wave. Only one study, which had a very limited data collection during the Omicron wave, suggested no difference in incidental versus attributable SARS-CoV-2 positivity between Delta and Omicron [15]. Due to this lack of data in the field and mixed reports on MIS-C differences during these waves, we chose to analyze our SARS-CoV-2 admissions with a thorough clinical chart review, including a three-person panel for adjudication, of admitted cases in a time period spanning the peaks of circulating Delta and Omicron strains in our community.

## 2. Methods

This retrospective cohort study is University at Buffalo Institutional Review Board approved #STUDY00005262. Patients were included in the acute COVID-19 analysis if they were ≤21 years of age and reported to have a positive diagnostic test for COVID-19. New York State strain surveillance data showed that after 1 August 2021, >97% of samples tested were Delta and by 1 January 2022, >89% of samples were Omicron/BA.1. BA.2/BA.2.12 were first noted in early March and rapidly increased to supplant BA.1. A nineteen-week period from August 1 to 12 December 2021 corresponded to a high proportion of Delta and captured the increase in regional hospitalizations [16]. A nine-week period from 26 December 2021 to 28 February 2022 were presumed to be due to the Omicron variant based on the same data set. Interim period cases (13–26 December) were not included in the analysis due to our inability to determine the causative variant. February 28 was chosen as the end point as the local BA.1-associated wave of increased hospitalizations was tapering off and state-wide variant data showed BA.2/BA.2.12 increasing in prevalence.

Adjudication of each PCR-positive case to ascribe if hospitalization was due to COVID-19 or for an unrelated disorder was performed by two independent reviewers (PK and AC). Disagreements were reviewed by a third reviewer who served as the tiebreaker (MH). Demographic data, presenting symptoms, findings, laboratory data, imaging, and assigned diagnoses, were tabulated. Groups were divided by causative variant as well as cases determined to be incidental, non-respiratory (but still due to COVID-19), or respiratory disease. For analysis of complexity of medical history, patients were classified as having no prior medical history, no serious medical history, or complex medical history using defined criteria including medical technology utilization as previously published [17].

Patients with MIS-C were excluded from the acute COVID-19 analysis only if they were not positive by PCR at the time of hospitalization. Patients with positive PCR at the time of concomitant MIS-C diagnosis (two in Delta, three in Omicron) were included in both analyses. Date of initial illness or positive molecular test was utilized to assign MIS-C patients to the correct wave. All patients who met criteria for MIS-C were reported to New York State on a prospective basis and also included in UB IRB STUDY00005262 prospectively.

Dichotomous variables were compared with Fisher’s exact test or chi squared (alpha of 0.05) to determine statistical significance. Continuous variables were evaluated with Student’s *t*-Test at 95% confidence and alpha of 0.05 to determine statistical significance. Vaccine efficacy (VE) was calculated using the formula VE = 1 − ARV/ARU where ARV is the attack rate in vaccinated patients (cases divided by vaccinated population) and ARU is the attack rate in unvaccinated patients (cases divided by unvaccinated population) [18].

## 3. Results

A total of 287 charts were evaluated, and 16 were omitted. Reasons included being out of the age range, records not associated with active COVID-19 cases, and duplicates. 17 charts (6.3%) required adjudication by the third reviewer (MDH) due to disagreements on inclusion. During the study period from 1 August 2021, through 28 February 2022, a total of 271 patients were admitted to our institution with the diagnosis of acute COVID-19. 16 patients admitted in the period of transition from Delta to Omicron were omitted from analysis [16]. Relevant patient characteristics are shown in Table 1.

Using zip code data from patient demographics to limit review to pediatric patients from Erie County (NY), during Delta, 4.6 patients were admitted to the hospital per 1000 PCR-positive cases in the community over the defined time period. During Omicron, this rate was 8.0 per 1000 cases. When only admissions attributed to COVID-19 were included, rates of admission in the Delta and Omicron waves were 3.8 and 5.9 per 1000 cases, respectively. Similar to previously reported data, the proportion of patients with race reported as Black was higher than was expected based on local demographic data. 25% of patients admitted during Delta and 24% of patients admitted during Omicron were Black compared to 10.8% of the general population in 2021 in our five-county catchment area [19]. This was statistically significant both during both waves with an odds ratio of admission for Black children testing positive with COVID-19 of 2.8 (1.3–6.0) during Delta and 2.5 (1.1–5.5) during Omicron.

Overall admissions in patients 0–4 years-of-age rose from 46% to 62% (*p* = 0.048) during Delta and Omicron, respectively. Admissions for the 12–18 age group fell from 31% to 17% of the total cohort (*p* = 0.04). The proportion of infants under two months of age, many of which underwent full sepsis workups due to neonatal fevers, was not significantly different between waves, making up 19% of those admitted during Delta compared to 14% during Omicron.

The association of medical history and admissions was assessed. Prior pulmonary disease (e.g., asthma or bronchopulmonary dysplasia) was similar between waves. Hospitalized patients during Omicron were more likely to have a history of seizures (1% vs. 11%, *p* = 0.02) or gastrostomy tube use (1% vs. 9%, *p* = 0.04) and less likely to have a history of diabetes (2% vs. 9%, *p* = 0.02). The contribution of complex medical history (or medical complexity [17]) to clinical presentation was evaluated. Proportions of these categories were similar between waves (Table 2).

Symptoms from patients’ histories were abstracted for analysis (Supplemental Table S1). The dominant symptoms were fever and respiratory complaints such as cough. Respiratory concerns such as congestion (68% vs. 52%, *p* = 0.064), sore throat (24% vs. 8%, *p* = 0.004), and dyspnea (54% vs. 37%, *p* = 0.03) were more prevalent during Delta. Gastrointestinal issues were the next most likely to be present. The remaining symptoms affected a minority of patients. Myalgias and headaches were more common during Delta. In contrast, rashes and hematologic derangements were more common during Omicron. Hematologic derangements included laboratory abnormalities such at elevated PTT, but also included severe anemia in 2 (1.7%) patients, thrombocytopenia in 6 (5.2%), and stroke in 1 (0.8%) patient. Of particular note, one patient during Omicron had otherwise unexplained new-onset, persistent vaginal bleeding that resolved as her COVID-19 symptomatology improved.

Overall, there were 13 coinfections noted during Delta (19%) and 18 noted in Omicron (16%, *p* = 0.54). A significantly higher percentage of respiratory cases during Delta had an identified coinfection (31% vs. 24%, *p* = 0.006). The vast majority of identified coinfections were respiratory viruses and occurred in patients admitted for respiratory support. One patient had port- and antibiotic-associated candidemia. There were no significant infectious complications noted after steroid administration.

In contrast with adult guidelines, while the majority of the patients admitted for acute COVID-19 received corticosteroids, only a minority received remdesivir. This usage was primarily limited to patients on non-invasive respiratory support, with only two patients without MIS-C or respiratory support receiving steroids and one patient without respiratory support receiving remdesivir. In total, 77% of patients with respiratory-dominant COVID-19 in Delta and 67% in Omicron received steroids (*p* = 0.26). In the same group, 38% received remdesivir in Delta compared to 12% during Omicron (*p* = 0.002).

Admission and discharge diagnoses (Table 3) were consistent with reported symptoms. Most commonly, patients were admitted for respiratory concerns, particularly pneumonia or bronchiolitis, reported as lower respiratory tract infection (LRTI). The diagnosis of croup was significantly more common during Omicron than Delta (*p* = 0.032). Dehydration without associated gastroenteritis and diabetic ketoacidosis (DKA) was significantly more common in Delta, though only in small numbers for each. Of the six total cases of DKA recorded in this cohort, only one was a new-onset case of diabetes, with the remainder occurring in patients with known Type 1 diabetes.

There were 29 patients with MIS-C admitted prior to Delta, between April 2020 and July 2021. Eleven patients were admitted with MIS-C following or during Delta infection, 3 during the transition period between Delta and Omicron, and 10 secondary to Omicron infection (Table 4). MIS-C rates fell with the transition from Delta (72 per 100,000 cases) to Omicron (49 per 100,000). MIS-C during Omicron showed reduced clinical severity in comparison to Delta, with statistically significant reduction in presence of shock, in length of stay, pressor requirements, and Intensive Care Unit (ICU) utilization. The majority of patients during the Omicron wave reported upper respiratory and gastrointestinal symptoms, though only cough and vomiting reached significance (Supplementary Table S2). During Omicron, cough was present in 60% (*p* = 0.02) and vomiting in 90% (*p* = 0.02) of cases.

Admission laboratory studies were evaluated for both patients with acute COVID-19 and patients with MIS-C (Table 5). Overall, all groups had elevated inflammatory markers. There were no statistically significant laboratory differences between the Delta and Omicron acute COVID-19 groups in laboratory values. Patients with MIS-C had more significant inflammatory markers, cardiac, hematologic, and electrolyte derangements than their acute COVID-19 counterparts.

There were no deaths recorded in this cohort. The readmission rate was low, with one patient in each of the acute COVID-19 and MIS-C groups requiring readmission.

Past immunity to SARS-CoV-2 was difficult to cleanly assess as most admitted patients did not have antibody testing and clear documented history of prior infections was infrequent. The Pfizer-BioNTech mRNA vaccine was available for 12 and older prior to the beginning of our study, and patients 5 to 11 years old became eligible during our study period prior to the Omicron wave. There was one previously vaccinated MIS-C patient during Delta and two during Omicron (one with two doses and one a single dose). Of the 55 (81%) patients with known vaccination status, none of those admitted with acute COVID-19 during Delta had been vaccinated. Of the acute COVID-19 admissions during Omicron, vaccine status was recorded in 88 of 107 (84%) of patients under the age of 18. Of these 88 patients, five (4%) were fully vaccinated and one (1%) had received a single dose. The remaining 82 (95%) were unvaccinated. From local county data, 33.0% of children under 18 were vaccinated as the Omicron wave began, and this increased to 42.1% by the end of the study period. Using this range, we calculated a vaccine efficacy in preventing hospitalizations of 87.8–91.7% when fully vaccinated and 85.1–89.9% for any vaccination (Supplementary Table S3).

## 4. Discussion

The in-depth evaluation described herein shows, despite a large number of incidental cases noted during the Omicron wave, there was an increased rate of pediatric hospitalizations. However, these patients were younger and less clinically severe than the prior Delta wave. Unfortunately, the effects of systemic racism continue to be felt as Black patients remain significantly more likely to be admitted. These findings mirror other recent publications utilizing large aggregated healthcare systems databases [20,21]. However, without the in-depth exploration of each case, those types of studies may overestimate the Omicron cases due to inclusion of a high number of incidental infections. Additionally, we chose to use PCR-based diagnosis to avoid inclusion of false positive cases.

It is clear that there remained significant pediatric morbidity even in what was the fourth large wave of COVID-19 in our geographic region. The proportion of children admitted in the 0–4 years-of-age group was significantly larger in the Omicron wave, in conjunction with a concurrent significant decrease in the 12–18 group. In a large population-based study in England, there was also noted a trend to a higher rate of admissions in young children during Omicron [4]. This is consistent with increased incidence of diagnoses affecting typically younger patients such as febrile seizures and croup. Laryngotracheitis has been described as a prominent feature of the Omicron variant’s infections in children in other studies [7,8].

As other studies support, acute cases during the Omicron wave were generally less severe. Our institution manages high flow nasal canula on medical-surgical wards so, as rates of pressor utilization were low outside of the MIS-C group, ICU utilization was limited. Respiratory support was required in a higher proportion by patients with complex medical history, which is a typical finding in this population with other respiratory viruses [5]. The study cohort had short stays and minimal repeat admissions related to either COVID-19 or MIS-C.

The detailed approach we undertook also helps to further elucidate how MIS-C is changing with time as we see fewer and less severe cases during Omicron. MIS-C made up 12% of hospital admissions during Delta compared to 6% during Omicron. Using prevalence of cases in our community, we estimate that the risk of MIS-C from Omicron is 32% lower (*p* = 0.04). A number of studies similarly report a lower incidence of MIS-C with Omicron, but these are few in number and focus on clinical presentations [9,10,11]. ICU admission and pressor use were higher in the pre-Delta and Delta MIS-C groups. Cardiac dysfunction was less common as well, though not statistically significant. Generally, length of stay was longer for children with MIS-C compared to acute COVID-19, and within MIS-C cases, the lengths of stay during Delta were also significantly longer. Our patients admitted with MIS-C were older than the average PCR-positive patient in both waves. In contrast to the patients with acute COVID-19, patients with MIS-C reported a narrower spectrum of symptomatology. Fever was always present and was most often accompanied by abdominal or upper respiratory symptoms, both of which were significantly more prevalent during Omicron. A portion of MIS-C patients in both waves had a history of rash or conjunctivitis, but no other typical features of Kawasaki Disease were noted, and no patients in this cohort met even incomplete criteria for this diagnosis. 

With the rise of Omicron, there were early reports of escape from neutralizing antibodies reported [22,23]. Population-based studies from Denmark [24] and England [4] support vaccine-derived protection in the Omicron wave. Patients aged 12 and above were eligible for vaccination during our entire study period. The age limit was reduced to include those 5 and above on 29 October 2021, as the local hospital volumes peaked with Delta-dominated cases. The estimated vaccine effectiveness of one dose of vaccine from our data is 88.5–90.6% for Omicron period, which is higher than some published estimates, but similar to a study from North Carolina [25]. In a large consortium of 31 United States hospitals, vaccine effectiveness during Omicron was estimated at 40% for adolescents and 68% for children 5–11 [26]. The overall protective effect of vaccination against hospitalization during Omicron in a 5–12 cohort in Singapore was estimated to be only 42.3% (95% CI, 24.9 to 55.7) [27]. Studies support that vaccine effectiveness wanes over time for Omicron [25,28]. Prior immunity may contribute to these disparate results in publications. As we had a robust Delta wave locally, this may have contributed to our estimate of vaccine effectiveness.

There are notable limitations to our study. First, we do not have direct sequencing data for every patient admitted to our institution, and a patient could be assigned to the incorrect variant. We limited this possibility by excluding patients with illness onset during the transition period between the Delta and Omicron waves and relied on New York State surveillance data to assign groups. Second, by only evaluating a single hospital, there are missed cases that could have affected results. Statewide data show that, while most patients in our geographic area ≤11 years of age were admitted to our hospital, a large proportion of patients ≥12 were not (Supplementary Table S4) [16]. This could be representative of hospitalizations that did not meet the threshold for a higher level of pediatric care, or it could represent referral to other tertiary centers given hospital crowding at the time. The single-center nature also provides relatively few patients for review. However, this study was designed to provide a thorough adjudicated review of cases, which would prove difficult in a larger study. Third, the retrospective nature of this report and the nature of our research focus shows that the evolution of the virus and its clinical presentation limit studies that claim to predict future outcomes.

## 5. Conclusions

This study provides a detailed look at patients admitted to a tertiary pediatric hospital during the Delta and Omicron waves of the ongoing COVID-19 pandemic. The data demonstrate statistically significant changes in the presenting symptoms as the variants evolved, with higher rates of croup and overall more diffuse symptomatology during the Omicron variant. Omicron caused less severe cases in both acute COVID-19 and MIS-C. This study adds to the literature supporting that vaccination protects from acute admissions and MIS-C, even into the Omicron wave. Since the symptomatology of presentations is evolving, the suspicion for COVID-19 as a causative agent in pediatric hospitalizations should remain high, even in patients without typical respiratory symptoms.

## Figures and Tables

**Table 1:** Characteristics of patients admitted with acute COVID-19 during the study period.

		Delta	Omicron	*p*-Value
	Total admitted (no.)	84	171	
	Due to COVID-19 [no. (%)]	68 (81%)	116 (66%)	0.05
	Non-respiratory [no. (%)]	29 (35%)	49 (28%)	0.96
	Respiratory [no. (%)]	39 (46%)	67 (38%)	0.96
Sex	Female [no. (%)]	30 (44%)	55 (47%)	0.69
	Male [no. (%)]	38 (56%)	61 (53%)	0.69
Race	White [no. (%)]	40 (59%)	62 (53%)	0.52
	Black [no. (%)]	17 (25%)	28 (24%)	0.90
	Other/Not declared [no. (%)]	11 (16%)	30 (26%)	0.14
Ethnicity	Hispanic [no. (%)]	4 (6%)	10 (9%)	0.50
Age	<2 months [no. (%)]	13 (19%)	16 (14%)	0.35
	0 to 4 [no. (%)]	31 (46%)	72 (62%)	0.048
	5 to 11 [no. (%)]	13 (19%)	20 (17%)	0.76
	12 to 18 [no. (%)]	21 (31%)	20 (17%)	0.04
	19 and above [no. (%)]	3 (4%)	4 (3%)	0.74
Duration of symptoms [Mean no. days (Median no. days)]		4.6 (3)	3.7 (2)	0.16
	Non-respiratory [Mean no. days (Median no. days)]	3.9 (2)	3.6 (2)	0.76
	Respiratory [Mean no. days (Median no. days)]	5.1 (4)	3.7 (2)	0.09
Length of stay [Mean no. days (Median no. days)]		3.7 (3)	2.8 (2)	0.12
	Non-respiratory [Mean no. days (Median no. days)]	2.6 (2)	2.2 (1)	0.64
	Respiratory [Mean no. days (Median no. days)]	4.5 (3)	3.1 (2)	0.12
Pressor Use [no. (%)]		4 (6%)	5 (4%)	0.64
	Non-respiratory [no. (%)]	3 (10%)	2 (4%)	0.36
	Respiratory [no. (%)]	1 (3%)	3 (4%)	1.00
NIPPV Use [no. (%)]		23 (34%)	23 (20%)	0.04
Ventilator Use [no. (%)]		1 (1%)	4 (3%)	0.43

**Table 2:** Past medical history in patients admitted with acute COVID-19.

	Delta			Omicron		
Type of diagnosis	No prior history	Limited history	Complex history	No prior history	Limited history	Complex history
Incidental [no. (%)]	9 (56.3%)	6 (38%)	1 (6.3%)	22 (40.0%)	22 (40%)	11 (20.0%)
Non-respiratory COVID-19 [no. (%)]	9 (30.0%)	13 (45%)	7 (23.3%)	30 (58.8%)	9 (18%)	10 (19.6%)
Respiratory COVID-19 [no. (%)]	15 (38.5%)	14 (36%)	10 (25.6%)	21 (30.4%)	21 (31%)	25 (36.2%)
Total COVID-19 [no. (%)]	35 (29.2%)	27 (40%)	17 (24.6%)	51 (42.5%)	30 (26%)	35 (29.2%)

**Table 3:** Discharge diagnoses for patients admitted with acute PCR-proven COVID-19, divided by system. Note that the total is greater than 100% due to some patients receiving more than one diagnosis due to complications of their acute infection.

Discharge Diagnosis	Delta	Omicron	*p*-Value
Pulmonary:			
Lower Respiratory Tract Infection [no. (%)]	31 (46%)	44 (38%)	0.352
URTI [no. (%)]	5 (7%)	2 (2%)	0.103
Croup [no. (%)]	2 (3%)	15 (13%)	0.032
Asthma exacerbation [no. (%)]	3 (4%)	7 (6%)	0.747
Apnea [no. (%)]	0 (0%)	1 (1%)	1.000
Inflammatory:			
Fever [no. (%)]	6 (9%)	5 (4%)	1.000
MIS-C [no. (%)]	2 (3%)	3 (3%)	1.000
Neonatal Fever/Sepsis Workup [no. (%)]	6 (9%)	9 (8%)	0.787
Cardiac:			
Cardiac arrest [no. (%)]	0 (0%)	2 (2%)	0.532
Myo/pericarditis [no. (%)]	0 (0%)	2 (2%)	0.532
Neurologic:			
TIA/Stroke [no. (%)]	1 (1%)	1 (1%)	1.000
Aseptic meningitis [no. (%)]	0 (0%)	1 (1%)	1.000
Seizure, including febrile seizure [no. (%)]	3 (3%)	9 (8%)	0.540
Altered mental status [no. (%)]	1 (1%)	0 (0%)	0.370
Gastrointestinal:			
Gastroenteritis [no. (%)]	7 (10%)	18 (16%)	0.378
DKA [no. (%)]	5 (7%)	1 (1%)	0.027
Dehydration/FTT [no. (%)]	5 (7%)	0 (0%)	0.006
Other:			
Inflammatory rash/urticaria [no. (%)]	1 (1%)	2 (2%)	1.000
Mucositis [no. (%)]	0 (0%)	1 (1%)	1.000
Rhabdomyolysis/myositis [no. (%)]	2 (3%)	2 (2%)	0.627
Acute chest crisis (in sickle cell disease) [no. (%)]	0 (0%)	3 (3%)	0.297
Vaso-occlusive crisis [no. (%)]	1 (1%)	2 (2%)	1.000
Vaginal bleeding [no. (%)]	0 (0%)	1 (1%)	1.000
Immune-mediated thrombocytopenia [no. (%)]	0 (0%)	1 (1%)	1.000
Chemotherapy complication [no. (%)]	0 (0%)	2 (2%)	0.532
Intra-uterine fetal demise [no. (%)]	1 (1%)	0 (0%)	0.370
Premature labor [no. (%)]	1 (1%)	1 (1%)	1.000

**Table 4:** Relevant characteristics of patients and their hospital stays while admitted with MIS-C temporally associated with the Delta and Omicron waves.

MIS-C Characteristics	Delta (*n* = 11)	Omicron (*n* = 10)	*p*-Value
Rate per 100,000 diagnosed cases of COVID-19 (Erie County, NY)	72	49	0.04
Age [years (95% CI)]	9.0 (2.9)	9.0 (3.4)	0.99
Female [no. (%)]	3 (29%)	4 (40%)	0.56
White [no. (%)]	4 (33%)	5 (50%)	0.56
Black [no. (%)]	5 (42%)	3 (30%)	0.50
Hispanic [no. (%)]	0 (0%)	2 (20%)	0.13
Prior ER visits [no. (%)]	1 (9%)	4 (40%)	0.11
Symptom duration [days (95% CI)]	3.9 (0.8)	4.1 (0.6)	0.67
Positive SARS-CoV2 PCR [no. (%)]	2 (17%)	3 (30%)	1.00
Length of Stay [days (95% CI)]	6.8 (2.0)	4.1 (0.7)	0.02
ICU stay [no. (%)]	10 (83%)	3 (30%)	0.01

**Table 5:** Laboratory data with mean values compared to normal ranges, contrasting variant-specific acute COVID-19 and MIS-C presentations.

		Delta		Omicron		*p*-Values for Specific Comparator Groups		
	normal range	Acute COVID-19	MIS-C	Acute COVID-19	MIS-C	MISC: Delta vs. Omicron	Delta: COVID-19 vs. MIS-C	Omicron: COVID-19 vs. MIS-C
Hemoglobin (g/dL) [mean (95% CI)]	11.5–14.5	13.5 (2.1)	11.2 (0.7)	12.1 (2.7)	11.4 (1.5)	0.82	0.000	0.312
White blood cell count (×10^9^/L) [mean (95% CI)] [mean (95% CI)]	4.0–12.0	9.8 (6.4)	10.6 (3.1)	10.1 (5.2)	11.5 (2.5)	0.62	0.660	0.305
Red blood cell count (10^12^/L) [mean (95% CI)] [mean (95% CI)]	4.0–5.3	4.8 (1.2)	4.2 (0.3)	4.5 (1.3)	4.2 (0.6)	0.97	0.009	0.351
Hematocrit [mean (95% CI)]	33.0–43.0	39.3 (7.7)	34.0 (2.4)	40.6 (32.8)	33.9 (4.5)	0.99	0.001	0.084
Neutrophil (count, ×10^9^/L) [mean (95% CI)] [mean (95% CI)]	1.5–6.6	6.8 (6.7)	8.6 (2.8)	5.9 (4.3)	8.7 (2.1)	0.95	0.267	0.015
Lymphocyte (count, ×10^9^/L) [mean (95% CI)]	1.0–5.5	2.5 (2.0)	1.1 (0.2)	3.0 (2.7)	2.3 (2.2)	0.18	0.000	0.479
Basophil (count, x10^9^/L) [mean (95% CI)]	<0.1	0.1 (0.7)	0.0 (0.0)	0.0 (0.0)	0.0 (0.0)	0.89	0.298	0.831
Eosinophil (count, ×10^9^/L) [mean (95% CI)]	<0.7	0.0 (0.1)	0.2 (0.2)	0.2 (0.8)	0.2 (0.1)	0.91	0.158	0.879
Monocytes (count, ×10^9^/L) [mean (95% CI)]	<1.0	0.9 (0.9)	0.4 (0.1)	0.9 (0.6)	0.8 (0.6)	0.17	0.000	0.706
Platelet (count, ×10^9^/L) [mean (95% CI)]	150–450	268.9 (112.3)	160.0 (38.3)	287.4 (130.7)	186.3 (67.7)	0.46	0.000	0.009
C-reactive protein (mg/L) [mean (95% CI)]	0.2–10.0	34.0 (41.0)	188.5 (53.6)	53.7 (97.3)	170.1 (169.4)	0.81	0.000	0.152
Erythrocyte sedimentation rate (mm/hr) [mean (95% CI)]	0–12	17.3 (17.2)	56.1 (20.0)	24.8 (24.5)	44.5 (22.9)	0.40	0.004	0.109
Lactate dehydrogenase (U/L) [mean (95% CI)]	155–345	560.8 (368.6)	451.6 (100.1)	891.5 (1988.8)	289.1 (47.1)	0.009	0.425	0.297
Ferritin (ug/L) [mean (95% CI)]	7–140	575.4 (1003.4)	1442.4 (1031.3)	923.9 (4744.3)	1056.1 (1007.0)	0.56	0.120	0.917
Albumin (g/dL) [mean (95% CI)]	3.5–5.0	3.9 (0.6)	3.5 (0.3)	4.7 (4.8)	3.8 (0.3)	0.13	0.041	0.171
Protein (g/dL) [mean (95% CI)]	4.8–7.8	6.7 (0.9)	6.5 (0.7)	6.8 (0.8)	6.7 (0.8)	0.76	0.594	0.714
Alanine Aminotransferase (u/L) [mean (95% CI)]	5–50	52.0 (92.8)	53.0 (31.6)	68.7 (304.4)	27.7 (11.2)	0.13	0.961	0.299
Aspartate Aminotransferase (U/L) [mean (95% CI)]	5–50	72.1 (119.7)	74.0 (42.6)	129.2 (479.5)	43.9 (30.6)	0.23	0.943	0.182
Alkaline Phosphatase (U/L) [mean (95% CI)]	80–345	167.8 (86.6)	164.7 (35.4)	205.7 (237.8)	166.3 (36.9)	0.94	0.884	0.271
Total bilirubin (mg/dL) [mean (95% CI)]	0.2–1.2	0.7 (1.0)	1.3 (1.1)	1.3 (3.2)	1.5 (0.7)	0.82	0.264	0.811
Sodium (mmol/L) [mean (95% CI)]	135–145	137.8 (3.4)	132.7 (2.0)	137.2 (4.2)	132.9 (2.0)	0.88	0.000	0.001
Potassium (mmol/L) [mean (95% CI)]	3.5–5.0	4.6 (0.8)	3.9 (0.5)	4.6 (0.8)	3.8 (0.2)	0.72	0.025	0.000
Chloride (mmol/L) [mean (95% CI)]	96–110	105.5 (4.3)	101.7 (3.0)	104.4 (4.2)	99.4 (4.2)	0.34	0.029	0.026
Bicarbonate (mmol/L) [mean (95% CI)]	20–30	18.8 (4.6)	16.8 (2.2)	19.5 (4.3)	28.4 (17.3)	0.17	0.116	0.273
Blood urea nitrogen (mg/dL) [mean (95% CI)]	5–19	10.9 (4.3)	14.0 (6.4)	12.0 (11.4)	15.2 (5.0)	0.75	0.328	0.231
Serum creatinine (mg/dL) [mean (95% CI)]	0.4–1.0	0.7 (0.3)	0.7 (0.2)	0.7 (0.9)	0.7 (0.1)	0.85	0.672	0.928
Institutional normalized ratio [mean (95% CI)]	N/A	1.1 (0.2)	1.4 (0.1)	1.2 (0.2)	1.3 (0.2)	0.13	0.000	0.119
Prothrombin time (seconds) [mean (95% CI)]	11.0–15.0	13.9 (1.6)	17.2 (1.2)	14.7 (1.8)	15.8 (1.5)	0.14	0.000	0.113
Partial thromboplastin time (seconds) [mean (95% CI)]	25.0–34.0	31.2 (4.7)	39.4 (9.0)	32.7 (6.5)	38.5 (3.1)	0.84	0.088	0.001
D-dimer (mcg/mL) [mean (95% CI)]	0.00–0.45	2.0 (3.3)	3.5 (1.6)	2.7 (5.1)	2.6 (1.3)	0.33	0.125	0.818
Fibrinogen (g/L) [mean (95% CI)]	200–470	377.3 (109.4)	626.6 (98.9)	420.5 (178.5)	608.8 (341.4)	0.90	0.001	0.222
Troponin-I (ug/L) [mean (95% CI)]	0.00–0.06	0.01 (0)	0.74 (0.82)	0.1 (0.1)	0.04 (0.04)	0.14	0.126	0.680
Brain natriuretic peptide (ng/L) [mean (95% CI)]	0–99	41.8 (50.8)	438.6 (274.9)	470.2 (1249.5)	85.9 (109.5)	0.011	0.004	0.227
Procalcitonin (mcg/L) [mean (95% CI)]	0.02–0.09	0.4 (0.8)	8.8 (10.3)	2.5 (11.9)	4.4 (2.7)	0.14	0.032	0.367

## Data Availability

Deidentified, summative data are available on request from the corresponding author.

## Supplementary Materials

The following supporting information can be downloaded at: https://www.mdpi.com/article/10.3390/v15010180/s1, Table S1: Symptoms and systems reported by patients and noted in chart as abnormal in patients admitted with acute COVID-19 (PCR-positive) during the Delta and Omicron waves. P-values comparing total prevalence to evaluate for statistically significant differences between the waves are also shown; Table S2: Symptoms and select findings reported by patients and select findings in those admitted with MIS-C temporally associated with the Delta and Omicron waves. (NOS: not otherwise specified; symptoms not easily categorized); Table S3: Calculation of vaccine efficacy, expanded; Table S4: Percent of patients hospitalized in (NYS portion of) catchment area who were admitted to our hospital during the study period. Note that our catchment area also extends to small portions of northwestern Pennsylvania which would reduce the proportion of patients admitted to our institution.
