# Supplementary material for: Decreased Clinical Severity of Pediatric Acute COVID-19 and MIS-C and Increase of Incidental Cases during the Omicron Wave in Comparison to the Delta Wave
Source: Viruses. 2023 Jan 7;15(1):180. doi: 10.3390/v15010180 (PMC9863387; doi:10.3390/v15010180)
Supplement: Supplementary file 1 [file viruses-15-00180-s001.zip › viruses-2098278-supplementary.pdf]

Supplementary Data:

Supplementary Table S1: Symptoms and systems reported by patients and noted in chart as abnormal in patients admitted with acute COVID-19 (PCR-positive) during the Delta and Omicron waves. P-values comparing total prevalence to evaluate for statistically significant differences between the waves are also shown.

	Delta			Omicron			
Symptom	Respiratory	Non-respiratory	Total	Respiratory	Non-respiratory	Total	p-values
Fever	24	19	43	48	37	85	0.1847
Conjunctivitis	1	3	4	1	3	4	0.4701
Rash	0	1	1	4	9	13	0.0189
Extremity changes	0	1	1	0	1	1	>0.9999
Adenopathy	1	1	2	1	4	5	>0.9999
Mucositis	0	0	0	0	4	4	0.2983
Cough	35	14	49	56	11	67	0.0588
Sore throat	13	3	16	5	4	9	0.0036
Congestion/Rhinorrhea	32	14	46	44	18	62	0.0644
Abdominal Pain	8	8	16	2	6	8	0.0025
Diarrhea	12	4	16	3	15	18	0.2373
Vomiting	14	16	30	18	22	40	0.2109
Arthritis	3	0	3	0	2	2	0.3603
Myalgia	8	4	12	1	7	8	0.0288
Chills	6	4	10	6	2	8	0.1213
SOB/inc WOB	34	3	37	44	0	44	0.0325
anosmia	2	2	4	1	1	2	0.1958
Headache	4	5	9	1	2	3	0.0099
AMS	0	2	2	2	2	4	>0.9999
Syncope	0	1	1	0	1	1	>0.9999
Shock (pressors)	1	2	3	2	2	4	0.7105
Derm NOS	3	2	5	5	8	13	0.4521
GI -NOS	2	6	8	3	2	5	0.0744
Respiratory -NOS	3	0	3	11	1	12	0.2633
CNS-NOS	1	5	6	7	14	21	0.1295
Renal	0	2	2	0	4	4	>0.9999
Cardiovascular - NOS	1	2	3	4	2	6	>0.9999
Hematologic	0	2	2	6	9	15	0.0325

Supplementary Table S2: Symptoms and select findings reported by patients and select findings in those admitted with MIS-C temporally associated with the Delta and Omicron waves. (NOS: not otherwise specified; symptoms not easily categorized)

	Delta	Omicron	p-value
Conjunctivitis	4 (36%)	5 (50%)	0.563
Rash	4 (36%)	6 (60%)	0.324
Adenopathy	2 (18%)	4 (40%)	0.296
mucositis	1 (9%)	4 (40%)	0.112
Cough	1 (9%)	6 (60%)	0.020
Sore throat	4 (36%)	3 (30%)	0.773
Congestion/Rhinorrhea	3 (27%)	3 (30%)	0.896
Abdominal Pain	4 (36%)	6 (60%)	0.324
Diarrhea	3 (27%)	6 (60%)	0.164
Vomiting	4 (36%)	9 (90%)	0.023
Myalgia	0 (0%)	3 (30%)	0.056
SOB	2 (18%)	1 (10%)	0.603
Headache	4 (36%)	2 (20%)	0.434
Shock (pressor requirement)	4 (36%)	0 (0%)	0.042
GI -NOS	1 (9%)	3 (30%)	0.240
CNS-NOS	0 (0%)	2 (20%)	0.126
CV - NOS or labs	7 (64%)	6 (60%)	0.881
Hematologic	7 (64%)	10 (100%)	0.058
Respiratory support	4 (36%)	1 (10%)	0.177
CXR abnormal	5 (45%)	2 (20%)	0.249
Left ventricular dysfunction	6 (55%)	2 (20%)	0.134

Supplementary Table S3: Values used for calculation of vaccine efficacy, expanded

	Vaccinated	Unvaccinated remainder	Total patients	% Vaccinated	% Unvaccinated
Fully vaccinated calculation	5	83	88	5.7%	94.3%
Calculation with any vaccination	6	82	88	6.8%	93.2%
	January, vaccinated at population-level	March, vaccinated at population-level			
Fully vaccinated calculation	0.33	0.421			
Calculation with any vaccination	0.33	0.421			
	Vaccine efficacy (low)	Vaccine efficacy (high)			
Fully vaccinated calculation	87.8%	91.7%			
Calculation with any vaccination	85.1%	89.9%			

Supplementary Table S4: Percent of patients hospitalized in (NYS portion of) catchment area who were admitted to our hospital during the study period. Note that our catchment area also extends to small portions of northwestern Pennsylvania which would reduce the proportion of patients admitted to our institution.

	Ages 0 to 4	Ages 5 to 11	Ages 12 to 18	Total
Hospitalized In Western NY (No.)	150	50	140	340
Hospitalized In Our Institution (No.)	133	49	80	262
Hospitalized In Our Institution (%)	89%	98%	57%	77%
